# A Needed Reform

**Published:** 1896-10

**Authors:** 


### A Needed Reform.

The Indiana State Board of Health has issued a circular letter to all railroad officials asking them to have ejected from their trains every man who persists in spitting on the floor of the cars or stations after he has been warned not to do so. In the circular the board explains that the sputum contains the germs of la grippe, nasal catarrh, and various other diseases. It also declares that "spitting is a nasty and unnecessary habit," and explains that the Board of Health will pass a rule against spitting which will have all the force of law if the railroads will post it up and endeavor to enforce it. The circular adds: "When the rule is first published and posted up in public places this board will, of course, be loudly abused as foolish, impracticable and idiotic. Attention thus being gained, we will publish in every county reason for the action." Such a reform as the Indiana health officers have undertaken is needed in every part of the United States.—*Chicago Medical Recorder.*

